# Alginates as food ingredients absorb extra salt in sodium chloride-treated mice

**DOI:** 10.1016/j.heliyon.2021.e06551

**Published:** 2021-03-23

**Authors:** Yukio Fujiwara, Ryoko Maeda, Hidenori Takeshita, Yoshihiro Komohara

**Affiliations:** a Department of Cell Pathology, Graduate School of Medical Sciences, Faculty of Life Sciences, Kumamoto University, Honjo 1-1-1, Kumamoto, 860-8556, Kumamoto, Japan; b Toy Medical Corporation, Saikumachi 4-40-1, Kumamoto, 860-0041, Kumamoto, Japan

**Keywords:** Alginates, Sodium chloride, Dietary fibres, Salt-sensitive hypertension

## Abstract

Many patients with impaired renal function undergoing dialysis are subject to severe dietary restrictions. Especially overdose of salt is related to crisis of their life, so their meals are basically salt-free or low salt. Therefore, their quality of life is declined due to their yearning for salty taste. In the present study, we searched new salt-adsorbing food materials in dietary fibers to develop food ingredients preventing salt-sensitive hypertension and kidney dysfunction. As a result, calcium alginate and ammonium alginate possessed sodium-binding capacity without releasing potassium which causes a problem in chronic kidney injury. Furthermore, the administration of those fibers inhibited blood NaCl concentration and induced NaCl excretion in mice model.

Therefore, calcium alginate and ammonium alginate are new candidate materials as salt-adsorbing materials, thus indicating that the health foods and/or health supplements containing those fibers may be a potentially new tool for prevention of salt-sensitive hypertension and kidney dysfunction.

## 1 Introduction

Over 1.1 billion people worldwide are estimated to suffer from hypertension, which is deeply involved in the onset and progression of cerebral cardiovascular disorders (Hay et al., 2020). The Guyton theory that sodium excretion failure in the kidney is necessary for the elevated blood pressure caused by salt intake is popular (Guyton, 1989). Renal sodium excretion failure induced by dysfunctions of the renin-angiotensin-aldosterone system and sympathetic nervous system causes salt-sensitive hypertension. In chronic kidney disease (CKD), the decline of renal function further induces dysfunction of renal sodium excretion (Kang et al., 2020; Cao et al., 2015; Johnson et al., 2014; Kimura et al., 1990).

Sodium accumulation triggers hypertension, followed by organ disorder. The renal dysfunction effect of sodium chloride has been demonstrated in an epidemiologic study (Verhave et al., 2004). Urinary albumin excretion correlates with urinary sodium excretion according to the results of 7,850 people in the Netherlands. These correlations are accepted even if they are corrected by sex, age, blood pressure, BMI, kidney function, blood glucose level, and smoking habits. This correlation is more strongly recognized being due to BMI increases (Verhave et al., 2004). In addition, there are several reports that a low salt diet is effective for disease prevention (Cook et al., 2007; Chaturvedi et al., 2011). Cerebral cardiovascular events after 10–15 years decreased by approximately 30% due to salt restriction for 18 months (Cook et al., 2007).

Therefore, food ingredients that suppress the absorption of sodium chloride are considered to be effective for preventing salt-sensitive hypertension. Seaweed, a traditional food, has various physiological functions. The water-soluble dietary fiber contained in seaweed can adsorb sodium in the gastrointestinal tract for excretion into faeces. Alginic acid is a food additive that has been used for a long time as a thickener, gelling agent and stabilizer in food. Alginic acid known as a dietary fiber is a polysaccharide that has a structure in which two types of uronic acids, such as mannuronic acid and guluronic acid, are linearly polymerized. The uronic acid constituting alginic acid has a carboxyl group with a high ion exchange property. Therefore, the carboxyl group of uronic acid can form a metal salt with an alkali metal ion, such as Na^+^ and K^+^. In addition, alginic acid can form metal salts with not only monovalent ions, such as Na^+^ and K^+^, but also multivalent ions, such as Ca^2+^.

Most of the alginate presently used in foods is sodium alginate, but potassium alginate, calcium alginate, and ammonium alginate are also used. Among them, potassium alginate has almost the same properties as sodium alginate and is therefore used as a thickener and gelling agent as a substitute for sodium alginate. A major characteristic of potassium alginate is its decomposition into alginic acid and potassium in the gastrointestinal tract. The separated alginic acid then combines with sodium in the gastrointestinal tract, followed by formation of sodium alginate and excretion from the body. Therefore, potassium alginate promotes excretions of hypertension-causing sodium (Tsuji et al., 1993). However, because potassium alginate releases potassium and does not adsorb sodium, accumulation of this released potassium is a problem in chronic kidney injury.

Thus, in the present study we searched for new salt-adsorbing food materials in dietary fibers, other than potassium alginate, to develop food ingredients preventing salt-sensitive hypertension and kidney dysfunction. Among 20 fibers, calcium alginate and ammonium alginate possessed the capacity to bind to sodium without releasing potassium. These fibers also reduced the blood NaCl concentration in the mouse model, suggesting that health foods and/or health supplements containing calcium alginate and ammonium alginate may potentially be a new tool for prevention of salt-sensitive hypertension and kidney dysfunction.

## 2 Materials and methods

### 2.1 Chemicals

Potassium alginate K-3, alginic acid, propylene glycol alginate, calcium alginate and ammonium alginate were purchased from the KIMICA Corporation (Tokyo, Japan). Gellan gum, guar gum, xanthane gum and diutan gum were purchased from Sansho Co., Ltd. (Osaka, Japan). Chitosan, laminaran and hydroxyethyl cellulose were purchased from Tokyo Chemical Industry Co., Ltd. (Tokyo, Japan). Polyacrylic acid, poly-γ-glutamic acid and methylcellulose were purchased from Wako Pure Chemical Industries Ltd. (Osaka, Japan). Sodium acrylate (polymer) was purchased from Nacalai Tesque Co., Ltd. (Kyoto, Japan). Glucomannan propol A, glucomannan leorex LM and glucomannan leorex RS were purchased from Shimazu Chemical Corporation (Hiroshima, Japan). Activated carbon was purchased from UES Co., Ltd. (Osaka, Japan). Carboxymethylcellulose sodium was purchased from Daicel FineChem Ltd. (Tokyo, Japan). Polystyrene sulfonic acid calcium was purchased from the NIPRO Corporation (Osaka, Japan). IPF-100K was purchased from ICHIMARU PHARCOS Co., Ltd. (Gifu, Japan). Amberlite was purchased from ORGANO Co., Ltd (Tokyo, Japan). All other chemicals were of the best grade available from commercial sources.

### 2.2 Effect of food materials on sodium absorption

Test materials were incubated in a 1% sodium chloride (NaCl) solution at room temperature for 10 min, followed by determination of the NaCl concentration using a salt meter (HORIBA Scientific, Model No. B-721).

### 2.3 Effect of food materials on potassium dissociation

Test materials were incubated in a 1% sodium chloride (NaCl) solution at room temperature for 10 min, followed by determination of the potassium concentration using a potassium meter (HORIBA Scientific, Model No. B-731).

### 2.4 Animals

C57BL/6N mice were obtained from CLEA Japan (Tokyo, Japan). Mice were housed in a temperature-controlled room with a 12-hour light/dark cycle. All animal experiments were approved by the Ethics Committee for Animal Experiments of Kumamoto University (H29-202) and were performed in accordance with the Guideline for Animal Experiments of the laboratories.

### 2.5 Effect of food materials on sodium absorption and potassium dissociation in mice

Mice were orally administered 12.5 or 25 mg of materials, followed by the oral administration of 1 mL of sodium chloride (10 mg/ml). Control mice were administered only sodium chloride. Blood samples were drawn from the orbital vein before and after administration. The Na concentration was measured using a Na and K meter, Fingraph (Otsuka Pharmaceutical Co., Lid, Tokushima, Japan) and the potassium concentration was measured using a Na and K meter, Fingraph (Otsuka Pharmaceutical Co., Lid, Tokushima, Japan). Mice were orally administered 12.5 or 25 mg of materials, followed by oral administration of 0.5 mL of sodium chloride (120 mg/ml). Faeces samples were dissolved in water, followed by determination of the Na concentration using a salt meter (HORIBA Scientific, Model No. B-721).

### 2.6 Measurement of alginate in the faeces

The content of alginate was measured colorimetrically by means of a naphtoresorcinol reaction (Momose et al., 1962). Briefly, dissolved solution of faece was added to 0.4% naphtoresorcinol solution and 8.5 M hydrochloric acid containing 0.05% copper sulfate. Then, the reaction mixture was heated in boiling water for 1 h, and the reaction products were extracted with butyl acetate. Absorbance of the extract was determined at 562 nm by a plate reader.

### 2.7 Measurement of sodium choloride in the faeces

The dissolved solution of faece was added in 0.5 M KCl solution to replace sodium alginate in faece into potassium alginate and NaCl. The NaCl concentration was measured using a salt meter (HORIBA Scientific, Model No. B-721).

### 2.8 Measurement of calcium in the blood and urine

The blood and urine samples were both reacted with reagents of calcium E-test kit (FUJIFILM Wako Pure Chemical Co., Osaka, Japan). The calcium concentration was determined by measuring absorbance at 610 nm by a plate reader.

### 2.9 Statistics

The data are expressed as the mean ± standard deviation (SD, n = 3–6 each groups). Differences between groups were examined for statistical significance using the Mann-Whitney U-test and non-repeated measures ANOVA. A value of *P* < 0.05 was considered statistically significant.

## 3 Results and discussion

### 3.1 Effect of test materials on the Na binding capacity

**Figure 1:**
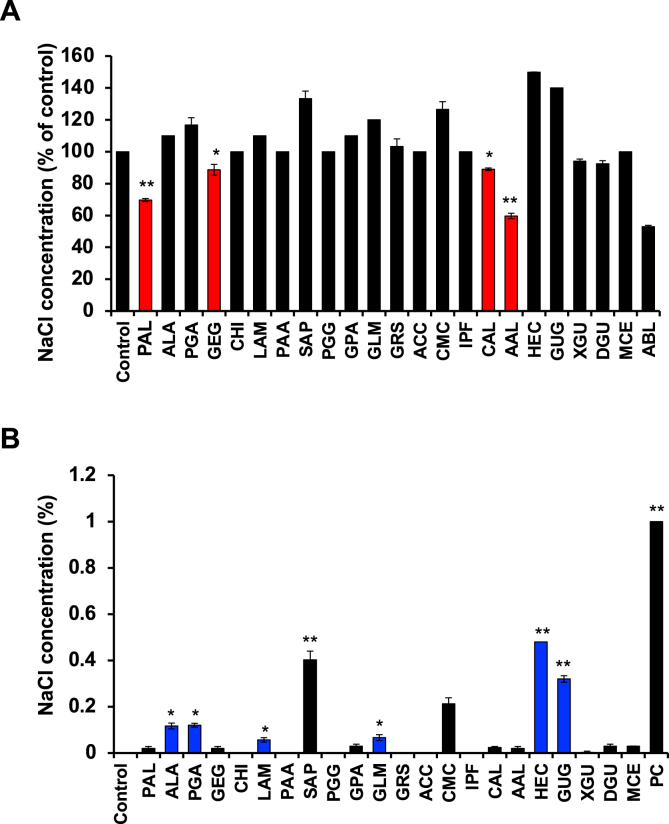
Effect of the test materials on the Na binding capacity. Test materials (50 mg/mL) (PAL, potassium alginate; ALA, alginic acid; PGA, propylene glycol alginate; GEG, gellan gum; CHI, chitosan; LAM, laminaran; PAA, polyacrylic acid; SAP, sodium acrylate (polymer); PGG, poly-γ-glutamic acid; GPA, glucomannan propol A; GLM, glucomannan leorex LM; GRS, glucomannan leorex RS; ACC, activated carbon; CMC, carboxymethylcellulose sodium; PSS, polystyrene sulfonic acid calcium; IPF, IPF-100K; CAL, calcium alginate; AAL, ammonium alginate; HEC, hydroxyetthyl cellulose; GUG, guar gum; XGU, xanthane gum; DGU, diutan gum; MCE, methylcellulose; ABL, amberlite) were incubated in a 1% NaCl solution (3 mL) for 10 min, then the NaCl concentration was determined, as described in the Materials and Methods (A) (n = 5 per group). Test materials (50 mg/mL) were incubated in a H_2_O solution (3 mL) for 10 min, then the NaCl concentration was determined, as described in the Materials and Methods (B) (n = 5 per group). One percent NaCl solution was used as a positive control (PC). The data are presented as the mean ± SD. ∗: p-value <0.01, ∗∗: p-value <0.001 vs. control.

**Figure 2:**
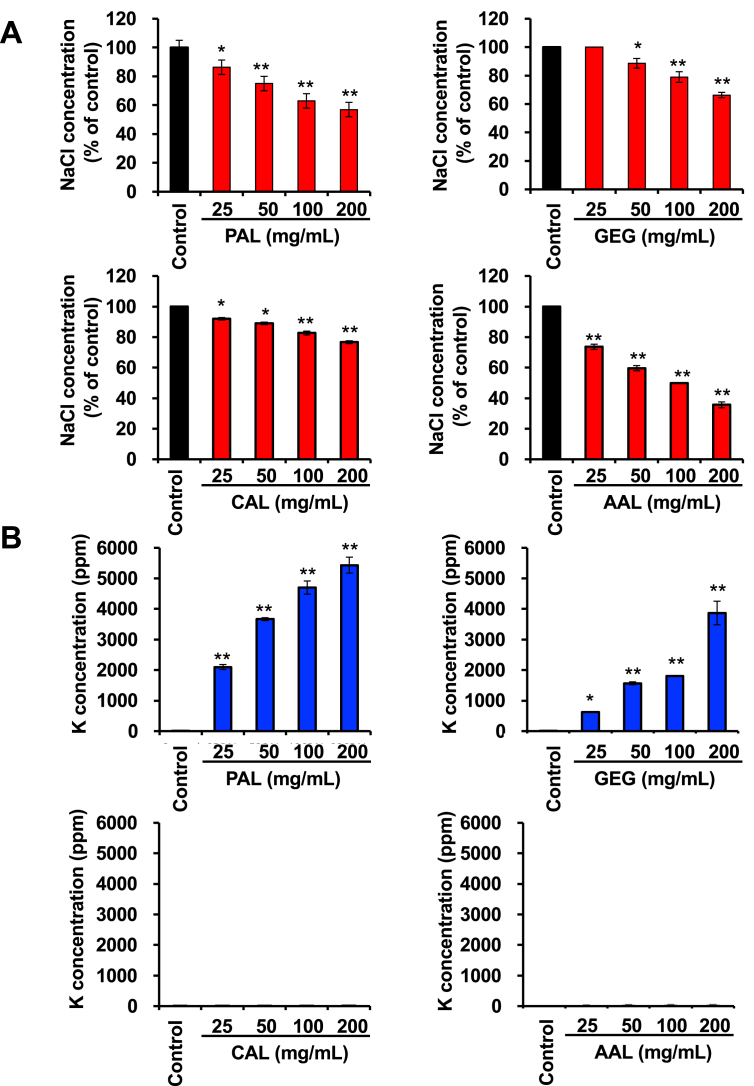
Effect of the test materials on the Na binding capacity and K release capacity. Test materials (25, 50, 100 and 200 mg/ml) were incubated in a 1% NaCl solution (3 mL) for 10 min, then the NaCl concentration was determined, as described in the Materials and Methods (A) (n = 5 per group). Test materials (25, 50, 100 and 200 mg/ml) were incubated in a 1% NaCl solution for 10 min, followed by determination of the potassium concentration, as described in the Materials and Methods (B) (n = 5 per group). Data are presented as the mean ± SD. ∗: p-value <0.05, ∗∗: p-value <0.001 vs. control.

**Figure 3:**
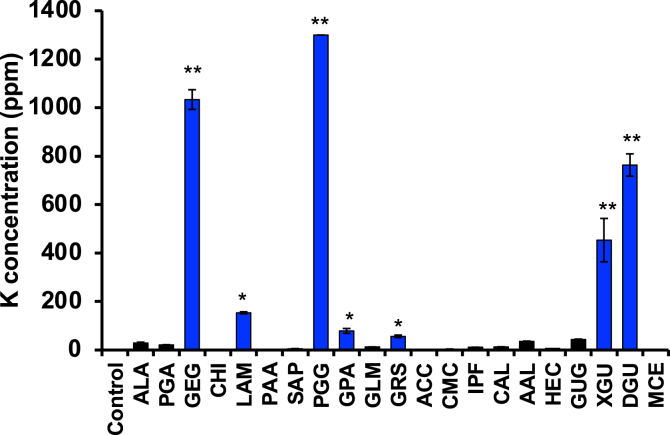
Measurement of containing potassium in test materials. Test materials (50 mg/mL) were incubated in a H_2_O solution for 10 min, then the K concentration was determined, as described in the Materials and Methods.

**Figure 4:**
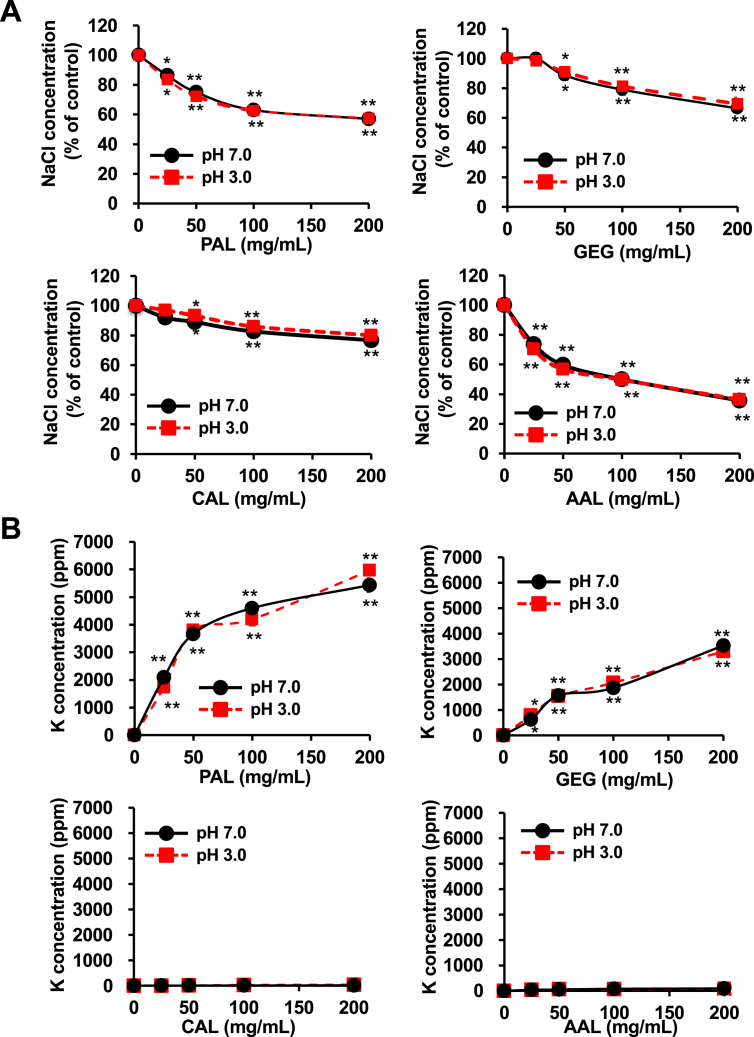
Effect of the test materials on the Na binding capacity and K release capacity under a strong acid condition. Test materials (25, 50, 100 and 200 mg/ml) were incubated in a 1% NaCl solution (3 mL) at pH 3.0 and pH 7.0 for 10 min, followed by determination of the NaCl concentration, as described in Materials and Methods. (A) (n = 5 per group). Test materials (25, 50, 100 and 200 mg/ml) were incubated in a 1% NaCl solution (3 mL) at pH 3.0 and pH 7.0 for 10 min, then the potassium concentration was determined, as described in the Materials and Methods (B) (n = 5 per group). Data are presented as the mean ± SD. ∗: p-value <0.05, ∗∗: p-value <0.001 vs. control.

In the present study, we chose dietary fibres as test materials. We first measured the effect of dietary fibres on the sodium binding capacity in a sodium chloride (NaCl) solution. As shown in Figure 1, amberlite (ABL), an ion exchange resin, used as a positive control significantly reduced the NaCl concentration in an NaCl solution. Similar results were observed with potassium alginate (PAL), gellan gum (GEG), calcium alginate (CAL) and ammonium alginate (AAL), indicating that these dietary fibers have the capacity to bind to Na. On the other hand, some test materials contained NaCl as an inclusion (Figure 1B). Furthermore, these identified dietary fibers reduced the NaCl concentration in a dose-dependent manner (Figure 2A). We also measured the effects of these identified dietary fibers on the potassium (K) concentration in solutions with or without NaCl. The potassium concentration was increased in a PAL and GEG-contained solution in a dose dependent manner but unaffected by CAL and AAL (Figure 2B), thus suggesting that PAL release potassium in addition to adsorbing sodium. On the other hand, it was also revealed that GEG has a sodium adsorbing function, whereas GEG contained potassium as an inclusion (Figure 3). In addition, we examined the effect on the NaCl and K concentrations under a strong acid condition. All of the identified fibers reduced the NaCl concentration, and PAL and GEG increased the potassium concentration in an NaCl solution under a strong acid condition (Figure 4A and 4B), indicating that the effects of these identified fibres on the Na binding capacity and potassium release capacity were maintained under a strong acid condition, such as in the stomach. Because the accumulation of potassium is a problem in chronic kidney injury, our data reveals that CAL and AAL are better dietary fibres in terms of sodium binding capacity than PAL and GEG.

### 3.2 Effect of test materials on the blood sodium and potassium concentrations in mice

**Figure 5:**
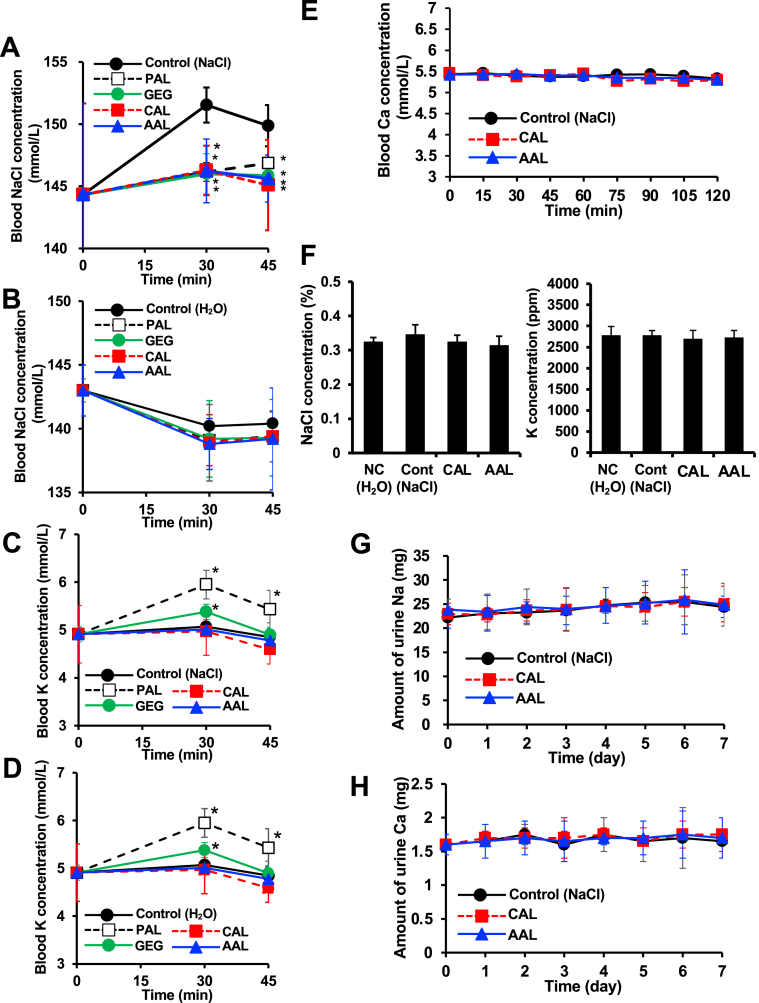
Effect of the test materials on the blood sodium and potassium concentration in sodium chloride-treated mice. Mice were orally administered 25 mg of PAL, GEG, CAL and AAL, followed by the oral administration of 1 mL of sodium chloride (10 mg/ml) (A) or 1 mL H_2_O (negative control) (B). Blood was collected after a predetermined time, then the NaCl concentration was determined, as described in the Materials and Methods (n = 5 mice per group). Mice were orally administered 25 mg of PAL, GEG, CAL and AAL, followed by the oral administration of 1 mL of sodium chloride (10 mg/ml) (C) or 1 mL of H_2_O (negative control) (D). Blood was collected after a predetermined time, then the potassium concentration was determined, as described in the Materials and Methods (n = 5 mice per group). Mice were orally administered 25 mg of CAL and AAL, and the blood was collected after a predetermined time, followed by the determination of the serum calcium concentration, as described in the Materials and Methods (E). Mice were orally administered 25 mg of CAL and AAL, followed by the oral administration of 0.5 mL of sodium chloride. Kidney was collected and homogenized after a predetermined time, then the NaCl concentration and K concentration were determined, as described in the Materials and Methods (F). Mice were orally administered 25 mg of CAL and AAL, followed by the oral administration of 1 mL of sodium chloride (10 mg/ml) every day for 1 week. The amount of sodium (G) and calcium (H) extraction in urine was determined described in the Materials and Methods. Data are presented as the mean ± SD. ∗: p-value <0.05 vs. control.

We next measured the effect of the candidate dietary fibres on the sodium binding capacity in mice. As shown in Figure 5A and 5B, the administration of NaCl significantly increased the blood concentration of NaCl in comparison to negative control (aqueous solution without NaCl). The administration of all candidate dietary fibers inhibited the increase of the blood NaCl concentration after the administration of NaCl (Figure 5A), whereas these fibers had no effect on the blood NaCl concentration without the administration of NaCl (Figure 5B). The inhibitory effects of CAL, AAL and GEG were as same as that of PAL (Figure 5A). The blood potassium concentration was not changed by the administration of CAL or AAL, but was increased upon administration of GEG and PAL with or without the administration of NaCl (Figure 5C and 5D). Furthermore, the serum calcium level was not changed after the administration of CAL and AAL (Figure 5E), suggesting that neither CAL nor AAL have an effect on the serum calcium concentration. Therefore, we selected CAL and AAL as better candidate fibres in terms of the sodium binding capacity. On the other hand, the renal sodium and potassium concentrations were both unchanged by the administration of sodium chloride, calcium alginate and ammonium alginate (Figure 5F), because this mouse model is not a chronic kidney injury model. In addition, when sodium chloride and alginates (CAL and AAL) were administered daily for one week, there was no change in the urinary sodium or calcium excretion in the 24-hour post-dose accumulation (Figure 5G and 5H).

### 3.3 Effect of CAL and AAL on sodium excretion in mice

**Figure 6:**
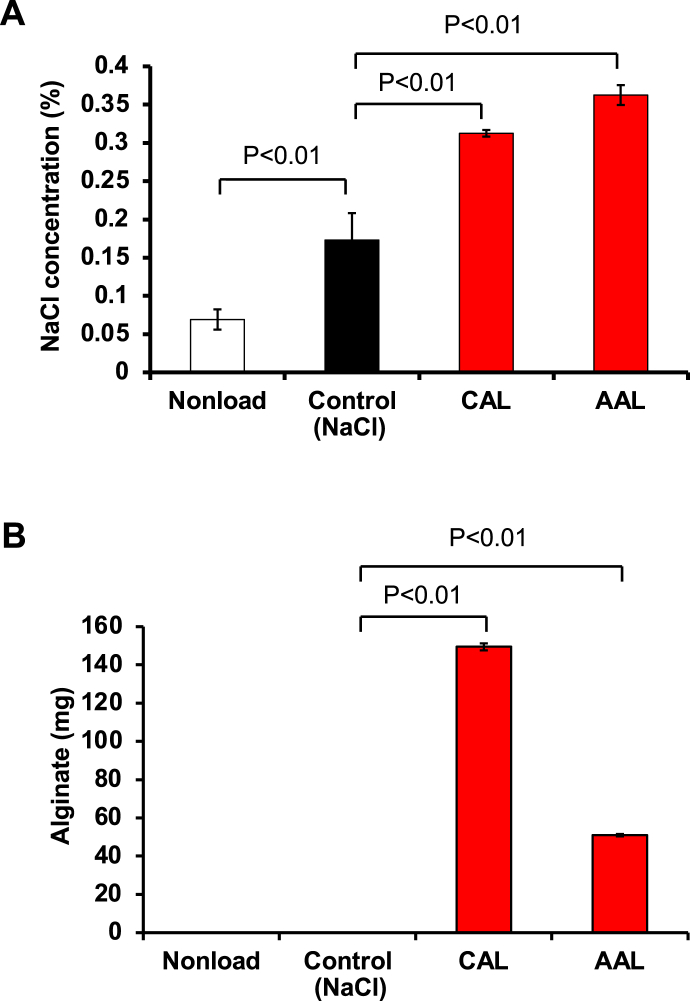
Effect of CAL and AAL on sodium excretion in sodium chloride-treated mice. Mice were orally administered 17.5 mg of CAL and AAL, followed by oral administration of 0.5 mL of sodium chloride (120 mg/ml). The faeces were collected after 24 h, followed by determination of the NaCl concentration (A) and alginate extraction (B), as described in the Materials and Methods (n = 5 mice per group).

We also checked the effect of CAL and AAL on sodium excretion in faeces. Administration of CAL and AAL led to increased sodium excretion into faeces (Figure 6A), and alginate was also detected in the faeces (Figure 6B), suggesting that the administered CAL and AAL bind sodium in digestive organs and that sodium-bound CAL and AAL are excreted in the faeces. Therefore, CAL and AAL are possible salt-adsorbing ingredients. It is known that potassium alginate decreases systolic blood pressure in deoxycorticosterone acetate (DOCA)-salt-induced spontaneous hypertensive rats (Chen et al., 2010) and that potassium alginate induces sodium excretion (Chen et al., 2010). In the present study, calcium alginate and ammonium alginate also have sodium binding capacity and a sodium excretion function. However, the present study yielded no direct evidence of the inhibitory effect of calcium alginate and ammonium alginate on hypertension. It is also reported that calcium alginate binds to cholesterol-derived bile acid, which is then excreted from the body and reduces the cholesterol concentration (Idota et al., 2016). Therefore, it is possible that calcium alginate induces the excretion of both sodium and cholesterol in the body. The present study demonstrated, for the first time, that the oral administration of ammonium alginate could decrease the serum sodium concentration and induce sodium extraction in mice. The rather short duration of calcium alginate and ammonium alginate treatment was a limitation of the present study. Thus, we cannot exclude the possibility that more prolonged calcium alginate and ammonium alginate treatment might show a greater benefit or that the beneficial effects of calcium alginate and ammonium alginate might be overcome by the effects of high salt intake. Further *in vivo* studies should be performed to assess the beneficial effects of calcium alginate and ammonium alginate in both mice and humans.

## 4 Conclusion

The present study reveals that calcium alginate and ammonium alginate are candidate salt-adsorbing ingredients other than potassium alginate, and they may lead to the resolution of problems in patients with chronic kidney injury. The findings indicate that health supplements that contain calcium alginate and/or ammonium alginate can be used to prevent salt-sensitive hypertension.

## Declarations

### Author contribution statement

Yukio Fujiwara, Ryoko Maeda: Conceived and designed the experiments; Performed the experiments; Analyzed and interpreted the data; Contributed reagents, materials, analysis tools or data; Wrote the paper.

Hidenori Takeshita, Yoshihiro Komohara: Conceived and designed the experiments; Contributed reagents, materials, analysis tools or data.

### Funding statement

This research did not receive any specific grant from funding agencies in the public, commercial, or not-for-profit sectors.

### Data availability statement

Data included in article/supplementary material/referenced in article.

### Declaration of interests statement

The authors declare no conflict of interest.

### Additional information

No additional information is available for this paper.
